# CRISPRpred(SEQ): a sequence-based method for sgRNA on target activity prediction using traditional machine learning

**DOI:** 10.1186/s12859-020-3531-9

**Published:** 2020-06-01

**Authors:** Ali Haisam Muhammad Rafid, Md. Toufikuzzaman, Mohammad Saifur Rahman, M. Sohel Rahman

**Affiliations:** 1 Department of Computer Science and Engineering, Bangladesh University of Engineering and Technology, Dhaka, Bangladesh; 2 Department of Computer Science and Engineering, United International University, Dhaka, Bangladesh

**Keywords:** CRISPR, sgRNA, Machine learning, Deep learning, Cas9

## Abstract

**Background:**

The latest works on CRISPR genome editing tools mainly employs deep learning techniques. However, deep learning models lack explainability and they are harder to reproduce. We were motivated to build an accurate genome editing tool using sequence-based features and traditional machine learning that can compete with deep learning models.

**Results:**

In this paper, we present CRISPRpred(SEQ), a method for sgRNA on-target activity prediction that leverages only traditional machine learning techniques and hand-crafted features extracted from sgRNA sequences. We compare the results of CRISPRpred(SEQ) with that of DeepCRISPR, the current state-of-the-art, which uses a deep learning pipeline. Despite using only traditional machine learning methods, we have been able to beat DeepCRISPR for the three out of four cell lines in the benchmark dataset convincingly (2.174%, 6.905% and 8.119% improvement for the three cell lines).

**Conclusion:**

CRISPRpred(SEQ) has been able to convincingly beat DeepCRISPR in 3 out of 4 cell lines. We believe that by exploring further, one can design better features only using the sgRNA sequences and can come up with a better method leveraging only traditional machine learning algorithms that can fully beat the deep learning models.

## Background

Genome editing has become extremely popular in recent times and more and more works are being done on it every day. One of the more widely used genome editing technologies is CRISPR-Cas9 (Clustered Regularly Inter-spaced Short Palindromic Repeats-CRISPR-associated protein 9). CRISPR-Cas9 is preferred over other technologies because of its higher degree of flexibility and accuracy in cutting and pasting genes. It is also more cost-efficient than other methods. Besides, it allows removing more than one gene at a time. By using CRISPR-Cas9, we are now able to manipulate multiple genes in plant and animal cells within weeks, which would otherwise have taken years before. Moreover, CRISPR-Cas9 can also edit genes of those species that were once considered resistant to genetic engineering.

### Motivation

Due to the off-target effect, the use of CRISPR-Cas9 on humans is still considered a risk [1]. Designing an accurate genome editing tool is hence, necessary. While designing such a tool, we only used sequence-based features. This is motivated by the empirical assertion of the natural belief (please see the recent Ph.D. thesis of Rahman [2] and the published results thereof in [3–5]) that the functional and structural information of a biological sequence are intrinsically encoded within its primary sequence. Also, recent works on this mainly leveraged deep learning techniques. Deep learning models are much harder to scale and reproduce. Furthermore, they are considered a black box because it is hard to explain what is happening inside a deep neural network. A recent study has analyzed several deep learning algorithms for top-n recommendation and concluded that most of the algorithms can be outperformed by simpler algorithms [6]. We maintain that the same can be done for CRISPR-Cas9 tools. Thus, besides trying to build an accurate tool using only sequence-based features, our main motivation was to build an easily reproducible and explainable traditional machine learning pipeline that can compete with state-of-the-art deep learning models.

### Previous works

In gene editing with CRISPR, we use a single guide RNA (sgRNA) with Cas-9 protein. The cut position in the DNA is specified by that sgRNA. Theoretically, we can engineer the sgRNA so that it binds to the site where it exactly matches the complement of the DNA strand. But in practice, cutting efficacy may vary significantly [7–9]. For this reason, predictive models are essential in designing sgRNAs.

There are many tools available for designing sgRNAs. These tools differ in the type of models used, selected features, genomes, etc. The tool sgRNA Designer [10] followed the rules proposed by Root laboratory [11]. Their training dataset contained genes from human and mouse cells. They used the Support Vector Machine (SVM) [12] classifier to select the best subset of features from among the 586 available features. Finally, a logistic regression model was trained for prediction [13]. Subsequently, this dataset was enriched and used by CRISPRpred [14], E-CRISPR [15], PROTOSPACER [16], CHOPCHOP [17] and WU-CRISPR [18]. Among these tools, CRISPRpred performed significantly better than others. The authors in [14], incorporated position-specific and position-independent features ranked by random forest [19, 20] and trained a SVM model for prediction.

DeepCRISPR [21] is the first tool to utilize deep learning for sgRNA activity prediction. It leveraged a deep unsupervised representation learning strategy to train a Deep Convolutional Denoising Neural Network (DCDNN) based Autoencoder [22] for learning features. These features are then fed into a Convolutional Neural Network (CNN) for training the prediction model. To evaluate DeepCRISPR, the authors in [21] have used a dataset comprising sgRNAs from four different cell types: HCT116, HEK293, HeLa and HL60. DeepCRISPR achieved an ROC-AUC score of 0.874, 0.961, 0.782 and 0.739 for the four cells respectively, beating the previous tools.

During the review and publication process of the current manuscript, a new deep learning based tool. called DeepHF [23], has been proposed. DeepHF uses a Bidirectional LSTM to extract features. Combining these features with hand-crafted biological features, a fully connected network has been trained to construct the final model. A dataset of about 50,000 sgRNAs with sgRNA activity for three SpCas9 variants was used for this model. It achieved Spearman correlation coefficients of 0.867, 0.862 and 0.860 for WT-SpCas9, eSpCas9(1.1) and SpCas9-HF1 respectively.

### Our contributions

In this paper, we present CRISPRpred(SEQ), a method to predict the on-target activities of sgRNAs using a traditional machine learning pipeline. A characteristic feature of CRISPRpred(SEQ) is that, unlike the previous models, it only focuses on sequence-based features. As mentioned before, we believe that the necessary information of a biological sequence can be decoded from its primary sequence. Indeed, our results in this research work further strengthen this assertion empirically as CRISPRpred(SEQ) has performed exceptionally well and has almost beaten the recent deep neural networking pipelines leveraging only traditional machine learning techniques and focusing only on primary sequence-based features. In particular, CRISPRpred(SEQ) has improved upon the results of DeepCRISPR by 2.174%, 6.905% and 8.119% for the cells HCT116, HeLa and HL60 respectively. Also, our preliminary experiments, without any hyperparameter tuning, achieved results close to that of DeepHF. This seems significant for at least two important reasons as follows. Firstly, this suggests that traditional machine learning techniques may have the potential to compete at par with the deep learning techniques and in a case like this, a simpler model is advantageous because of its scalability and interpretability. And secondly, this further suggests that our (human) feature engineering exercise, as has been done in case of CRISPRpred(SEQ), which is an integral step in any traditional machine learning pipeline, has almost been able to beat automatic feature extraction of the deep networking pipelines.

## Results

### DeepCRISPR dataset

**Fig. 1:**
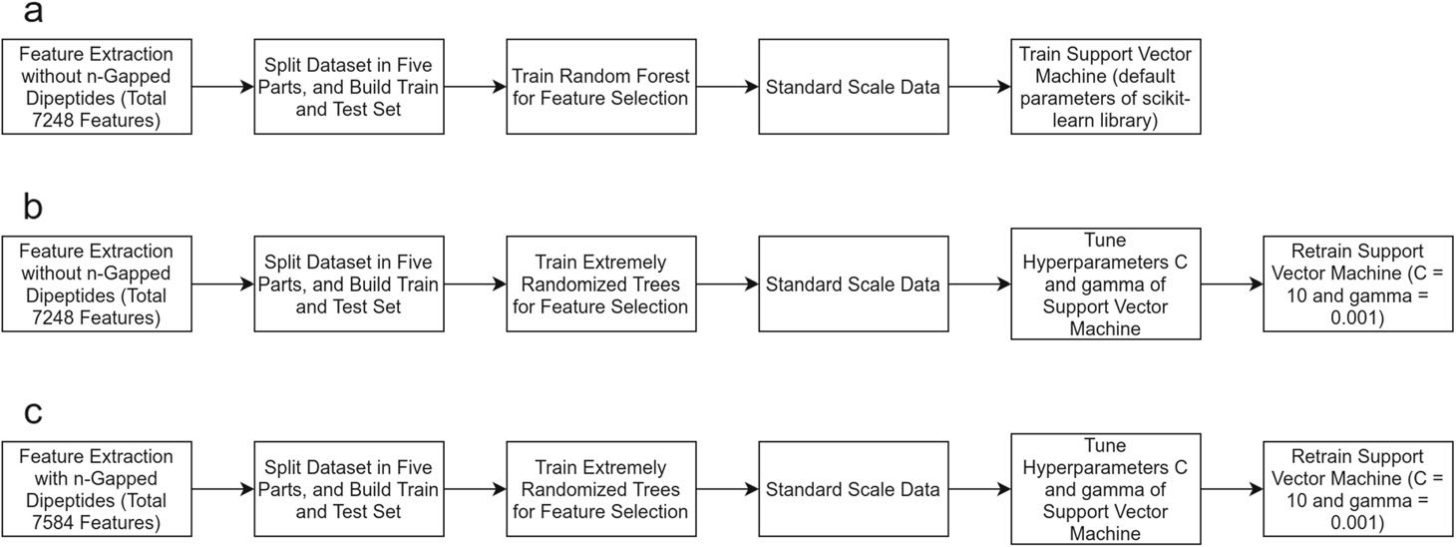
Training pipelines, the steps of building the final prediction model. **a***The pipeline for experimental setup A.* We only extracted position-independent and position-specific features. The steps of splitting the dataset and selecting features are described in “Results” section. We used the default parameters while training SVM. **b***The pipeline for experimental setup B.* The steps of extracting features and splitting dataset is same as experimental setup A. But, in feature selection step we used extremely randomized trees (the feature selection criteria are described in “Results” section). We performed hyperparameter tuning on SVM and retrained SVM with the best hyperparameters. **c***The pipeline for experimental setup C.* It is exactly same as the pipeline for experimental setup B except we considered the feature type n-Gapped Di-nucleotide in feature extraction step

We have used the on-target dataset used in [21] (i.e., for DeepCRISPR), which was also used by Haeussler et al. [24]. This whole dataset contains a total of over 16,000 labeled sgRNAs for four cell types, namely, HCT116, HEK293, HeLa and HL60 (Please check Additional file 1, 2, 3 and 4, respectively). We carried out our experiments in 6 different settings (A, B, C, D, E and F) to be described below. The experimental pipelines are shown in Fig. 1.

We split the dataset for four cells separately in 5 parts where the splits were stratified by data labels. For experimental setup A, B and C, we selected one of the five parts for each cell as test dataset (20% of the data) and the combination of remaining four parts for each cell as the training dataset(80% of the data). This was done for all the five parts and every experiment was done five times for each testing and training datasets combination. We, however, did not remove the common data between training and testing datasets. The authors of DeepCRISPR also did not remove the common data between training and testing datasets in their first experiment. Although they removed it in their later experiments, they pretrained their model with a large number of unlabeled data and from the description of the data it seemed that there are common portions between the labeled and unlabeled data.

However, for experimental setup D, E and F, we ignored the data for HEK293 cell due to the below par results achieved in experiments A, B and C for this cell. We performed leave-one-cell-out experiments where we chose one of the five parts for one cell as the testing dataset and the remaining four parts for the remaining cells as the training dataset. For example, if we chose to leave out the data for HCT116 from the training dataset, we chose one of the five parts for the same as the testing dataset and selected the remaining four parts for HeLa and HL60 and combined them to be used as the training dataset.

### Results of experimental setup a

In this setting, we have used the training data to train the pipeline used in CRISPRpred [14] (CRISPRpred(SEQ)-A). We have extracted position-independent and position-specific features (“Feature extraction” section) and then, used random forest to rank the features using Gini score [25] and then selected the top 2899 features (number of features used in [14]). Then support vector machine (with default parameters of scikit-learn [26] library) was used to train the final model.

**Fig. 2:**
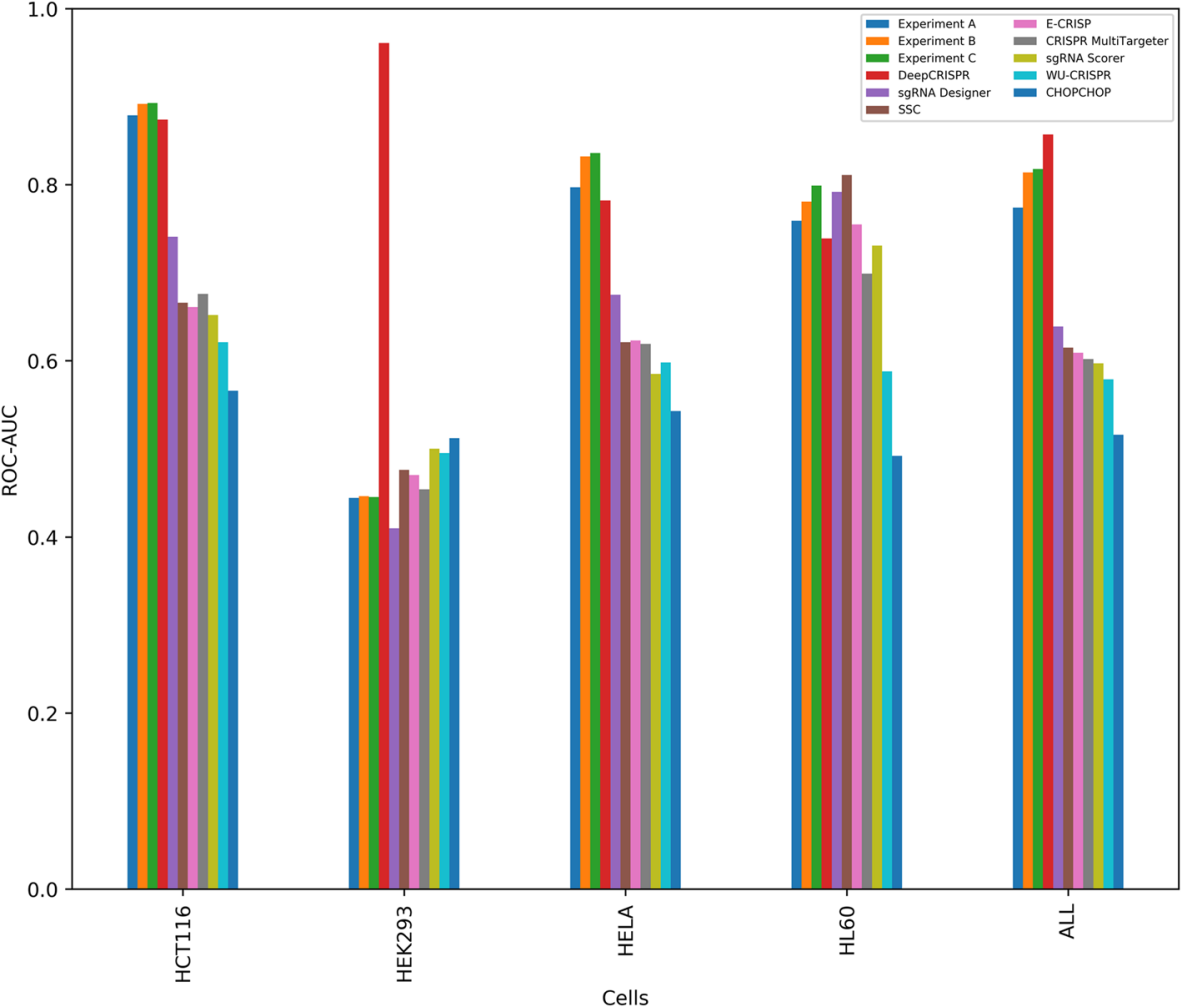
Comparison of performance of various methods with first three experimental settings (A, B and C). Y-axis denotes the ROC-AUC and X-axis denotes the cell types. In all three settings, CRISPRpred(SEQ) has convincingly beaten DeepCRISPR in 3 out of 4 cells, i.e., in HCT116, HeLa and HL60. However, in HEK293, DeepCRISPR performs far better than CRISPRpred(SEQ) (please also see a relevant discussion in “Results on HEK293 cell” section). CRISPRpred(SEQ)-C performs slightly better than CRISPRpred(SEQ)-B which in turn outperforms CRISPRpred(SEQ)-A in all cell lines

The results were compared with DeepCRISPR [21], sgRNA Designer [10], SSC [27], CHOP-CHOP [17], CRISPR MultiTargeter [28], E-CRISP [15], sgRNA Scorer [29], Cas-Designer [30] and WU-CRISPR [18] (Fig. 2). Following the paper presenting DeepCRISPR [21], we have used ROC-AUC as the metric for comparison.

From the results we observe that the state-of-the-art tool, DeepCRISPR, has achieved an ROC-AUC score of 0.874, 0.961, 0.782, 0.739 for the cells HCT116, HEK293, HeLa and HL60 respectively whereas CRISPRpred(SEQ)-A has achieved an ROC-AUC score of 0.879, 0.444, 0.797 and 0.759 for the four cells respectively (Fig. 2, Additional file 5). Thus, CRISPRpred(SEQ)-A was able to beat DeepCRISPR for 3 out of 4 cells.

### Results of experimental setup b

In this setting, unlike the normal CRISPRpred pipeline, we used extremely randomized trees [31] instead of the random forest to rank and select the features (the difference between random forest and extremely randomized trees is discussed in “Extremely randomized trees vs random forest” section). Like before, the features were extracted and then ranked using the Gini score. Then we selected the features having a score greater than or equal to the mean Gini score, thereby selecting around 1995 features. Then we performed 3 fold cross-validation on the training data for tuning the SVM parameters *C* and *γ*. Increasing the value of *γ* means that SVM tries more to exactly fit the training data. On the other hand, the *C* parameter controls the smoothness of the decision boundary. We tuned our model for *C* values of 1, 10 and 100 and *γ* values of 0.0001, 0.001, 0.01 (Table 1). We achieved the best cross-validation result for *C*=10 and *γ*=0.001. We determined the best hyperparameters based on ROC-AUC.

**Table 1:** The results of 3 fold cross-validation hyperparameter tuning of Experiment B

*γ*	0.0001	0.001	0.01
*C*			
1	0.702	0.775	0.759
10	0.733	0.781	0.758
100	0.765	0.781	0.758

All the values in the table are ROC-AUC. The best result has been achieved for *C*=10 and *γ*=0.001

Subsequently, we retrained the model (CRISPRpred(SEQ)-B) using the best hyperparameters and then have compared the results with the previous tools as is done in Experiment A (Fig. 2, Additional file 5). CRISPRpred(SEQ)-B has achieved an ROC-AUC of 0.892, 0.446, 0.832 and 0.781 for the cells HCT116, HEK293, HeLa and HL60 respectively, improving further upon the results of CRISPRpred(SEQ)-A.

### Results of experimental setup c

In this setup, we added a new feature type called n-gapped di-nucleotide (“Feature extraction” section) along with the previous features used in Experiments A and B. Similar to the previous pipeline, then the features were ranked and selected using extremely randomized trees (features having a score greater than or equal to the mean Gini score), thereby selecting a total of around 1957 features. We again performed 3 fold cross-validation to tune the SVM parameters *C* and *γ* (Table 2). After the final training of the model (CRISPRpred(SEQ)-C) with the best hyperparameters (*C*=10 and *γ*=0.001), we compared the results with the previous tools (Fig. 2, Additional file 5). CRISPRpred(SEQ)-C registered a slight improvement over CRISPRpred(SEQ)-B with an ROC-AUC of 0.893, 0.445, 0.836 and 0.799 for the cells HCT116, HEK293, HeLa and HL60 respectively, which might be attributed to the newly added n-gapped di-nucleotide features.

**Table 2:** The result of 3 fold cross-validation hyperparameter tuning of Experiment C

*γ*	0.0001	0.001	0.01
*C*			
1	0.705	0.783	0.763
10	0.732	0.788	0.762
100	0.763	0.788	0.762

All the values in the table are ROC-AUC. The best result is achieved for *C*=10 and *γ*=0.001

### Results of experimental setup d, e and f

**Fig. 3:**
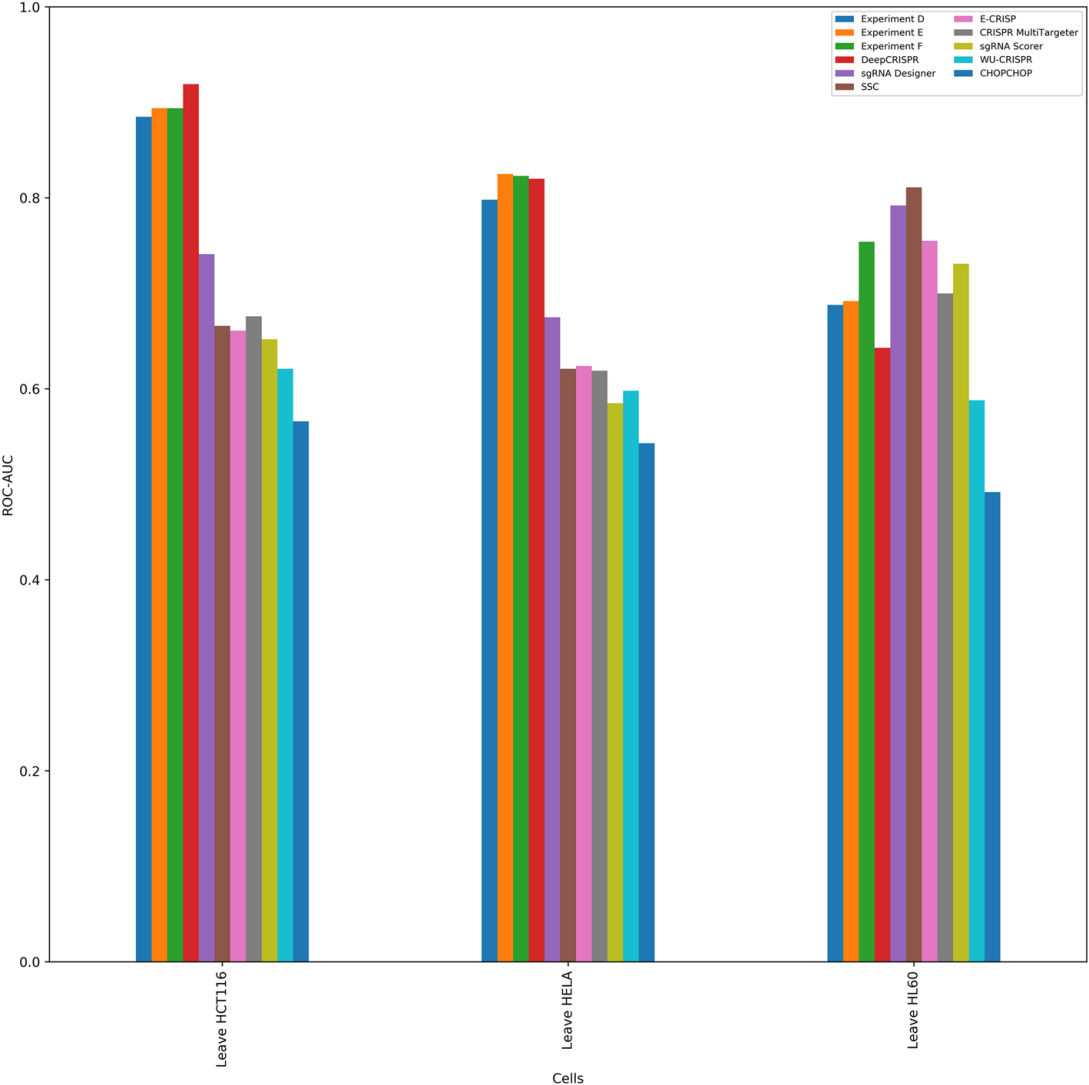
Comparison of performance of various methods with the three experimental settings D, E and F. Y-axis denotes the ROC-AUC and X-axis denotes the cell type that we are leaving out. In all three settings, CRISPRpred(SEQ) has beaten DeepCRISPR when leaving out cell HL60. CRISPRpred(SEQ)-E and CRISPRpred(SEQ)-F achieved scores slightly better than DeepCRISPR when leaving out cell HeLa. None of the settings were able to beat DeepCRISPR when leaving out cell HCT116 but managed to achieve a score close to that of DeepCRISPR

We followed the exact pipeline for A, B and C for Experiments D, E and F respectively but we did not perform any hyperparameter tuning for Experiments E and F. For Experiments E and F, we used the best hyperparameters (*C*=10 and *γ*=0.001) found in Experiments B and C and for Experiment D, we used the default parameters as was done in Experiment A. We then compared the results with the same previous tools that we used to compare in Experiments A, B and C. Recall that in these experiments we did leave-one-cell-out cross validation excluding HEK-293 cell from the experiments (a discussion on HEK-293 cell data is provided in “Discussion” section). DeepCRISPR achieved ROC-AUC values of 0.919, 0.82 and 0.643 while leaving out cells HCT116, HeLa and HL60 respectively. In Experiment D we achieved an ROC-AUC score of 0.885, 0.798 and 0.688 while leaving out cells HCT116, HeLa and HL60 respectively, only managing to beat DeepCRISPR when leaving out HL60. In Experiment E we were able to improve the result of experiment D further by achieving an ROC-AUC scores of 0.894, 0.825 and 0.692 respectively outperforming DeepCRISPR when leaving out cells HeLa and HL60. There was a noticeable improvement when leaving out HL60 in Experiment F. Here we managed to attain ROC-AUC values of 0.894, 0.823 and 0.754 while leaving out cells HCT116, HeLa and HL60 respectively. All the detailed comparisons are provided in Additional file 6 and illustrated in Fig. 3.

### Preliminary experiments on deepHF dataset

As has already been mentioned above, during the review and publication process of the current manuscript a new tool named DeepHF has been reported in [23]. We repeated the experimental setups A, B and C on the DeepHF dataset used in [23] (without any hyperparameter tuning). This dataset contains over 50,000 sgRNAs, covering approximately 20,000 genes with sgRNA activities for three SpCas9 variants, namely WT-SpCas9, eSpCas9(1.1) and SpCas9-HF1 (Additional file 7). We used the same pipelines used in Experiments A, B and C except that, in this case regression models for tree models and SVM were used. The dataset was split into training (85% of the data) and testing datasets (15% of the data). For experiment A, we achieved Spearman correlation coefficients of 0.829, 0.814 and 0.804 for WT-SpCas9, eSpCas9(1.1) and SpCas9-HF1 respectively. Spearman correlation coefficients were 0.837, 0.825 and 0.816 for WT-SpCas9, eSpCas9(1.1) and SpCas9-HF1 respectively in Experiment B. There was a small improvement in Experiment C with Spearman correlation coefficients of 0.838, 0.830 and 0.821 for WT-SpCas9, eSpCas9(1.1) and SpCas9-HF1 respectively. We managed to be very close to the correlation coefficients achieved by DeepHF which are 0.867, 0.862 and 0.860 for WT-SpCas9, eSpCas9(1.1) and SpCas9-HF1 respectively. The authors of [23] did not use SVM because it took a long time to finish. But, we managed to use an SVM library that utilized the GPU (ThunderSVM [32]) and our preliminary results strongly suggest that we can achieve a greater coefficient than DeepHF if we run a hyperparameter tuning algorithm with more hyperparameters in consideration.

## Discussion

### Traditional machine learning vs deep learning

CRISPRpred(SEQ) has performed remarkably well for 3 out of 4 cells for the DeepCRISPR dataset thereby (almost) beating a deep learning pipeline (DeepCRISPR) leveraging only classical machine learning methods. A simpler traditional machine learning model is useful because- i) it is easier to reproduce the results of a traditional machine learning pipeline, ii) it is faster to compute, iii) deep learning models are more prone to overfitting because of their large hypothesis space [33], iv) and they are hardly interpretable.

### Extremely randomized trees vs random forest

One of the main reasons behind using extremely randomized trees is that it is much faster than random forest (about three times faster) [31] given that one of our goals is to build a simpler and faster model. Unlike random forest models, extra trees algorithm selects splits totally or partially at random [31].

### Results on HEK293 cell

The performance of CRISPRpred(SEQ) on HEK293 cell is not up to the mark; thus a brief discussion on this point is in order. The dataset for HEK293 cell had been termed as an outlier in prior literature [24] because of being responsible for a lot of off-targets. Also, they have a very high GC contents. Further details with statistics can be found in [24]. So, the quality of dataset for HEK293 cell might not be as good as the datasets for other cells. We have noticed that almost all the previous tools also did not perform well for this cell.

At this point, we also would like to remark on the (excellent) result of DeepCRISPR for the HEK293 cell line. As has been mentioned above, DeepCRISPR has first leveraged a deep unsupervised representation learning strategy to train a DCDNN based Autoencoder [22] for learning features. Here they have used over 70GB of unlabeled data by generating all 23 nucleotide sequences ending with NGG, NAG, CCN and CTN from the Human genome directly and then using ENCODE data to add epigenomic information to each record [34]. This resulted in about 0.68 billion unlabeled sgRNA sequences. Now, this raises the question of whether extra bias was introduced given that overlapping sgRNAs between test data and unsupervised data were not removed.

### Sequence based features

We note that, apart from some important changes in the machine learning pipeline employed, CRISPRpred(SEQ) principally differs from its predecessor, CRISPRpred, in that the former only focuses on sequence-based features whereas the latter have considered other types of features as well. This focus on only sequence-based features was motivated by the empirical assertion of the natural belief [2–5]) that the functional and structural information of a biological sequence are intrinsically encoded within its primary sequence. We believe that further research along this line is in order from both pure biological (to validate this hypothesis) and computational biology (to propose and evaluate more tools based on only sequence-based features) point of view.

### DeepHF dataset

We have only done preliminary experiments with the DeepHF dataset. This dataset consists of over 50,000 sgRNAs which requires more computational time to train. Although we managed to find an SVM library that uses GPU, we had to use a library for tree algorithms that uses CPU. So, we decided not to perform hyperparameter tuning for the DeepHF dataset. We also believe that considering more values for hyperparameters *C* and *γ* of SVM and also adding hyperparameters such as the number of trees in a random forest and extremely randomized trees, the number of features selected during hyperparameter tuning can improve our model further.

## Conclusion

CRISPRpred(SEQ) has performed exceptionally well and has almost beaten the deep neural networking pipelines leveraging only traditional machine learning techniques and focusing only on primary sequence-based features. In particular, CRISPRpred(SEQ) has improved upon the results of DeepCRISPR by 2.174%, 6.905% and 8.119% for the cells HCT116, HeLa and HL60 respectively. We believe, that we can improve our model if we have more computational resources which will allow us to explore our model further.

## Methods

### Feature extraction

sgRNA sequence consists of four types of nucleotides: Adenine (A), Cytosine (C), Thymine (T) and Guanine (G). We extracted three types of features related to the composition of nucleotides in the sgRNAs. Two types of features were already used in CRISPRpred [14]. We added a new type of feature called n-gapped di-nucleotide which is similar to the feature n-gapped dipeptide used in [35]. Nevertheless, the features are described below for the sake of completeness.

- **Position Independent Features (PIF):** These features represent the number of occurrences of a given n adjacent nucleotides in the entire sequence. We extracted PIF features for *n*=1,2,3,4.
  For example, the feature named AC indicates how many times the sequence AC appears in a given sgRNA sequence. For the sgRNA
  $$\textbf{AC}\text{ATCAGGTT}\textbf{AC}\text{CTCT}\textbf{AC}\text{CAAGG}$$
  the number of times AC appears is 3.
  The number of PIF when *n*=1 is 4, namely A, C, T, G. In the same way, number of PIF when *n*=2 is 4^2^, i.e., AA, AC, …, GG. The number of PIF when *n*=3 and *n*=4 is 4^3^ and 4^4^ respectively. Thus, the total number of PIF is
  $$4^{1}+4^{2}+4^{3}+4^{4}=340$$
- **Position Specific Features (PSF):** This type of features are all binary features indicating whether a nucleotide or n adjacent nucleotides appear at a certain position in a given sgRNA. Again, we varied n from 1 to 4 for generating PSF.
  The feature named AA_2_ indicates whether the sequence AA appears at position 2 or not for a sgRNA. For the sgRNA
  $$\mathrm{G}\textbf{AA}\text{ACAGGAGGCGGTAAAGGAGG}$$
  the value of AA_2_ is 1.
  When *n*=1, there are 23 possible positions in which 4 possible nucleotides can appear. For *n*=2, there are 22 possible positions in which 4^2^ possible 2 adjacent nucleotides can appear. In the same way, for *n*=3, the number of possible positions for 4^3^ possible 3 adjacent nucleotides to appear is 21. 4^4^ possible 4 adjacent nucleotides can appear at 20 possible positions. So, number of PSF, considering 1≤*n*≤4 is
  $$4\times 23 + 4^{2} \times 22 + 4^{3} \times 21 + 4^{4} \times 20=6908$$
- **n-Gapped Di-nucleotides (nGD):** This type of features were used in [35]. In this type, we counted the number of times 2 given nucleotides appear at a certain distance in a sgRNA.
  The feature named GAP:AG_2_ specifies the number of times A and G occur at a distance of 2 nucleotides with order of nucleotides preserved. In other words, the value of GAP:AG_2_ may not be equal to the value of GAP:GA_2_. For the sgRNA,
  $$\text{AGATTCTTTGG}\mathbf{A\underline{TC}G}\mathrm{G}\mathbf{A\underline{GG}G}\text{AGG}$$
  the value of GAP:AG_2_ is 2.
  2 specific nucleotides can appear at a distance of 1, 2, 3,…, 21. Possible combination of 2 nucleotides is 4^2^. The total number of nGD is
  $$4^{2}\times21=336.$$

### Feature selection

We extracted a total of 7584 features. A large number of features have a chance of over-fitting the model. Also, it increases the training time. To reduce the dimension of feature space, first, we ranked the features using random forest (for experiments A and D) and extremely randomized trees (for experiments B, C, E and F) using the Gini score. The number of estimators while training random forest and extremely randomized trees was 500.

### Standard scaling

First, we scaled all of our features using standard scaling. Standard scaling is a data pre-processing technique. It transforms data so that the mean of all data is 0 and the standard deviation is 1. Among the extracted features there are binary features like if a nucleotide is present or absent at a specific position. These types of features can only be equal to 0 or 1. There are also some features like how many times a nucleotide occurs in the entire sequence. These features’ values have a different domain than binary features. So the feature values have varying ranges. Several machine learning models assume that the features have been scaled to the same value range. If there is a feature with a higher variance compared to other features then it will dominate the objective function and the model will not be able to learn from other features.

### Support vector machine (SVM)

We experimented with several supervised machine learning algorithms to train our model. Finally, a nonlinear support vector machine (SVM) was used to train our final model. We used radial basis function (RBF) as the kernel for SVM. We also tuned the hyperparameters *C* and *γ* which has already been described. Finally, we trained our model for *C*=10 and *γ*=0.001.

### Experimental environment

We have conducted experiments using python language (version 3.6). We mainly used the scikit-learn package (version 0.20.3) [26] of python for all machine learning related programming. Pandas (0.24.2) and NumPy (1.16.2) library was used for data management. We later used Thundersvm [32] to train SVM which makes use of GPU.

The experiments were carried out on Kaggle Kernel and in a server machine. Kaggle is a cloud computational environment. It had 4 CPU cores with 17 Gigabytes of RAM. In a single session, it provides 9 hours of execution time with 5 Gigabytes of auto-saved disk space and 16 Gigabytes of temporary disk space. The server machine was equipped with Intel Xeon CPU E5-4617 @ 2.90GHz x 6, Ubuntu 13.04 64-bit OS and 64GB RAM.

Later, we used a machine equipped with NVIDIA GP102 Titan Xp graphics processor, Intel Core i5-8400 CPU @ 2.80GHz x 6, Ubuntu 18.04 64-bit OS and 16GB RAM to run the experiments for DeepHF feature.

## Supplementary information

**Additional file 1** This file contains the DeepCRISPR data for cell HCT116.

**Additional file 2** This file contains the DeepCRISPR data for cell HEK293.

**Additional file 3** This file contains the DeepCRISPR data for cell HeLa.

**Additional file 4** This file contains the DeepCRISPR data for cell HL60.

**Additional file 5** This file contains the detailed comparison of experimental setup A, B and C with previous tools.

**Additional file 6** This file contains the detailed comparison of experimental setup D, E and F with previous tools.

**Additional file 7** This file contains the dataset for DeepHF.

## Data Availability

The datasets generated and/or analysed during the current study are available in the CRISPRpredSEQ repository, https://github.com/Rafid013/CRISPRpredSEQ The codes to reproduce the results of the experiments are also available in the same repository.

## Abbreviations

CRISPR: Clustered regularly inter-spaced palindromic repeats
Cas9: CRISPR associated protein 9
sgRNA: Single guide ribonucleic acid
DCDNN: Deep Convolutionary Denoising Neural Network
CNN: Convolutional Neural Network
SVM: Support vector machine
ROC: Receiver operating characteristic
AUC: Area under the curve
PIF: Position independent features
PSF: Position specific features
nGD: n-gapped di-nucleotides

## References

[1] Rubeis G, Steger F (2018). Risks and benefits of human germline genome editing: An ethical analysis. Asian Bioeth Rev.

[2] Rahman MS (2018). Sequence based computational methods for protein attribute prediction and phylogeny reconstruction. PhD thesis.

[3] Rahman MS, Rahman MK, Saha S, Kaykobad M, Rahman MS (2019). Antigenic: An improved prediction model of protective antigens. Artif Intell Med.

[4] Rahman MS, Rahman MK, Kaykobad M, Rahman MS (2018). isgpt: An optimized model to identify sub-golgi protein types using SVM and random forest based feature selection. Artif Intell Med.

[5] Rahman MS, Shatabda S, Saha S, Kaykobad M, Rahman MS (2018). Dpp-pseaac: A dna-binding protein prediction model using chou’s general pseaac. J Theor Biol.

[6] Dacrema M. F., Cremonesi P., Jannach D.Are we really making much progress? a worrying analysis of recent neural recommendation approaches. In: Proceedings of the 13th ACM Conference on Recommender Systems. ACM: 2019. 10.1145/3298689.3347058.

[7] Jinek M, Chylinski K, Fonfara I, Hauer M, Doudna JA, Charpentier E (2012). A programmable dual-rna–guided dna endonuclease in adaptive bacterial immunity. Science.

[8] Shalem O, Sanjana NE, Hartenian E, Shi X, Scott DA, Mikkelsen TS, Heckl D, Ebert BL, Root DE, Doench JG (2014). Genome-scale crispr-cas9 knockout screening in human cells. Science.

[9] Wang T, Wei JJ, Sabatini DM, Lander ES (2014). Genetic screens in human cells using the crispr-cas9 system. Science.

[10] Doench JG, Fusi N, Sullender M, Hegde M, Vaimberg EW, Donovan KF, Smith I, Tothova Z, Wilen C, Orchard R (2016). Optimized sgrna design to maximize activity and minimize off-target effects of crispr-cas9. Nat Biotechnol.

[11] Cui Y, Xu J, Cheng M, Liao X, Peng S (2018). Review of crispr/cas9 sgrna design tools. Interdiscip Sci Comput Life Sci.

[12] Cortes C, Vapnik V (1995). Support-vector networks. Mach Learn.

[13] Pei Z, Liu J, Liu M, Zhou W, Yan P, Wen S, Chen Y (2018). Risk-predicting model for incident of essential hypertension based on environmental and genetic factors with support vector machine. Interdiscip Sci Comput Life Sci.

[14] Rahman MK, Rahman MS (2017). Crisprpred: A flexible and efficient tool for sgrnas on-target activity prediction in crispr/cas9 systems. PloS one.

[15] Heigwer F, Kerr G, Boutros M (2014). E-crisp: fast crispr target site identification. Nat Methods.

[16] MacPherson CR, Scherf A (2015). Flexible guide-rna design for crispr applications using protospacer workbench. Nat Biotechnol.

[17] Labun K, Montague TG, Gagnon JA, Thyme SB, Valen E (2016). Chopchop v2: a web tool for the next generation of crispr genome engineering. Nucleic Acids Res.

[18] Wong N, Liu W, Wang X (2015). Wu-crispr: characteristics of functional guide rnas for the crispr/cas9 system. Genome Biol.

[19] Ho T. K. (1995). Random decision forests. Proceedings of 3rd International Conference on Document Analysis and Recognition, vol. 1.

[20] Ho T. K. (1998). The random subspace method for constructing decision forests. IEEE Trans Pattern Anal Mach Intell.

[21] Chuai G, Ma H, Yan J, Chen M, Hong N, Xue D, Zhou C, Zhu C, Chen K, Duan B (2018). Deepcrispr: optimized crispr guide rna design by deep learning. Genome Biol.

[22] Schmidhuber J (2015). Deep learning in neural networks: An overview. Neural Netw.

[23] Wang D, Zhang C, Wang B, Li B, Wang Q, Liu D, Wang H, Zhou Y, Shi L, Lan F (2019). Optimized crispr guide rna design for two high-fidelity cas9 variants by deep learning. Nat Commun.

[24] Haeussler M, Schönig K, Eckert H, Eschstruth A, Mianné J, Renaud J-B, Schneider-Maunoury S, Shkumatava A, Teboul L, Kent J (2016). Evaluation of off-target and on-target scoring algorithms and integration into the guide rna selection tool crispor. Genome Biol.

[25] Gini C. In: Pizetti E, Salvemini T, (eds).Variabilità e mutabilità (variability and mutability). 1955 ed. Bologna, Reprinted in Memorie di metodologica statistica. Rome: Libreria Eredi Virgilio Veschi ; 1912.

[26] Pedregosa F, Varoquaux G, Gramfort A, Michel V, Thirion B, Grisel O, Blondel M, Prettenhofer P, Weiss R, Dubourg V, Vanderplas J, Passos A, Cournapeau D, Brucher M, Perrot M, Duchesnay E (2011). Scikit-learn: Machine learning in Python. J Mach Learn Res.

[27] Xu H, Xiao T, Chen C-H, Li W, Meyer CA, Wu Q, Wu D, Cong L, Zhang F, Liu JS (2015). Sequence determinants of improved crispr sgrna design. Genome Res.

[28] Prykhozhij SV, Rajan V, Gaston D, Berman JN (2015). Crispr multitargeter: a web tool to find common and unique crispr single guide rna targets in a set of similar sequences. PloS one.

[29] Chari R, Mali P, Moosburner M, Church GM (2015). Unraveling crispr-cas9 genome engineering parameters via a library-on-library approach. Nat Methods.

[30] Park J, Bae S, Kim J-S (2015). Cas-designer: a web-based tool for choice of crispr-cas9 target sites. Bioinformatics.

[31] Geurts P, Ernst D, Wehenkel L (2006). Extremely randomized trees. Mach Learn.

[32] Wen Z, Shi J, Li Q, He B, Chen J (2018). ThunderSVM: A fast SVM library on GPUs and CPUs. J Mach Learn Res.

[33] Russell S, Norvig P (2009). Artificial Intelligence: A Modern Approach, 3rd edn..

[34] Chuai G.Private Communication. 2019.

[35] Rahman MS, Rahman MK, Kaykobad M, Rahman MS (2018). isgpt: An optimized model to identify sub-golgi protein types using svm and random forest based feature selection. Artif Intell Med.

